# Characterization and Quantification of Compounds in the Hydroalcoholic Extract of the Leaves from *Terminalia catappa* Linn. (Combretaceae) and Their Mutagenic Activity

**DOI:** 10.1155/2014/676902

**Published:** 2014-03-09

**Authors:** Francisco José Mininel, Carlos Sérgio Leonardo Junior, Lívia Greghi Espanha, Flávia Aparecida Resende, Eliana Aparecida Varanda, Clarice Queico Fujimura Leite, Wagner Vilegas, Lourdes Campaner dos Santos

**Affiliations:** ^1^Organic Chemistry Department, Institute of Chemistry, São Paulo State University (UNESP), 14800-900 Araraquara, SP, Brazil; ^2^Department of Biological Sciences, Faculty of Pharmaceutical Sciences, São Paulo State University (UNESP), 14801-902 Araraquara, SP, Brazil; ^3^São Paulo State University (UNESP), Experimental Campus of São Vicente, 11350-000 São Vicente, SP, Brazil

## Abstract

*Terminalia* is a genus of Combretaceous plants widely distributed in tropical and subtropical regions. Thus, the aim of this study was to quantify the majority compounds of the hydroalcoholic extract (7 : 3, v/v) of the leaves from *T. catappa* by HPLC-PDA, chemically characterize by hyphenated techniques (HPLC-ESI-IT-MS^n^) and NMR, and evaluate its mutagenic activity by the *Salmonella*/microsome assay on *S. typhimurium* strains TA98, TA97a, TA100, and TA102. The quantification of analytes was performed using an external calibration standard. Punicalagin is the most abundant polyphenol found in the leaves. The presence of this compound as a mixture of anomers was confirmed using HPLC-PDA and ^1^H and ^13^C NMR. Mutagenic activity was observed in strains TA100 and TA97a. As the extract is a complex mixture of punicalagin, its derivatives, and several other compounds, the observed mutagenicity may be explained in part by possible synergistic interaction between the compounds present in the extract. These studies show that mutagenic activity of *T. catappa* in the Ames test can only be observed when measured at high concentrations. However, considering the mutagenic effects observed for *T. catappa*, this plant should be used cautiously for medicinal purposes.

## 1. Introduction

As part of our efforts to identify the substances responsible for the pharmacological activities of plants utilized in Brazilian popular medicine (Biota-Fapesp Project number 09/52237-9), we have analyzed the hydroalcoholic extract of the leaves from* Terminalia catappa* L., which belongs to the family Combretaceae. The trivial names of these plants in Brazil include “amendoeira-da-praia,” “cuca,” and “chapéu de sol.”

In several Asian countries, physicians have used the leaves, bark, and fruit of* T*.* catappa* to treat dermatitis and pyresis [1–4]. Moreover, the antimicrobial and antifungal [5–8], antioxidative [9–11], anti-inflammatory [12, 13], hepatoprotective [3, 4], antidiabetic [14], carcinogenesis-preventing effects [15–17], antimalaric [18], and antinociceptive [19] of* T. catappa* could potentially provide benefits to human health.

Natural products have also been isolated from the plant* T. catappa* which contains triterpenoids (ursolic acid, asiatic acid), squalene but no caffeine, flavonoids (isovitexin, vitexin, and rutin), gallic acid, hydrolysed tannins such as punicalagin anomers as a major component, punicalin, terflavins A and B, tergallagin, tercatain, chebulagic acid, geranin, granato B, and corilagin [13, 20, 21].

Tannins have been reported to be responsible for decreases in food intake, growth rate, feeding efficiency, net metabolizable energy, and protein digestibility in experimental animals [21]. Incidences of certain cancers, such as esophageal cancer, have been reported to be related to consumption of tannin-rich foods such as betel nuts and herbal teas, suggesting that tannins might be carcinogenic [22]. However, other reports have indicated that the carcinogenic activity of tannins might be related to components that are associated with tannins, rather than the tannins themselves [23, 24]. Tannins have also been reported to exert other physiological effects, such as the acceleration of blood clotting, a reduction in blood pressure, a decrease in serum lipid levels, liver necrosis, and modulation of the immune response [21, 25].

Ellagitannins have been isolated along with gallotannins from various other plant families and show little chemotaxonomic significance. The tannins punicalagin and punicalin, both of which contain a gallagyl unit and were first isolated from the pericarps of pomegranate (*Punica granatum*, Punicaceae) [26], were a characteristic component in some* Terminalia* species. This supports the hypothesis that Punicaceae is chemotaxonomically proximate to Combretaceae as classified by Cronquist [27].

Despite its biological properties, the chemical composition may vary according to geographiclocation [28, 29], a fact impairing the standardization of raw material and commercial products for medicinal purposes [30].

Considering that there are no data in the literature on quantification of the majority secondary metabolites of the hydroalcoholic extract of the* T. catappa* by HPLC-PDA and the popular use of this plant, in this study the punicalagin anomers and ellagic acid were isolated and quantified.

Moreover, the mutagenic effects were evaluated using a* Salmonella*/microsome assay (Ames test) in the presence and in the absence of* in vitro *metabolizing systems in the concentrations 22.24, 16.68, 11.12, 5.56, and 2.78 mg/plate.

## 2. Experimental

### 2.1. Chemical and Reagents

HPLC-grade methanol (MeOH) and acetonitrile were purchased from J.T. Baker (Baker-Mallinckrodt, Phillipsburg, NJ, USA). Standards of ellagic acid were purchased from Sigma Chemical Co. (St. Louis, MO, USA) and had a purity of 95%. The anomers mixture of punicalagin *α* and *β* isolated of the hydroalcoholic extract from* T. catappa* (with 98% purity) were used as external standards. HPLC-grade water (18 MΩ·cm) was obtained using a direct Milli-Q purification system (Millipore Co., Bedford, MA, USA). Sep-Pak RP18 cartridges (500 mg/mL) for solid-phase extraction (SPE) were purchased from Phenomenex Co. (Torrance, CA, USA).

### 2.2. Apparatus

The HPLC system used was a JASCO 2010 HPLC (Jasco, Tokyo, Japan) equipped with PU-2089S Plus pump, a MD-2018 Plus Photodiode Array Detector (PDA), an AS-2055 Plus auto sampler, and column oven (CO-2065 plus). The software ChromNav (Workstation JASCO-ChromNav 1.18.03) was used to control the analytical system and data collection and processing. To purify the substances, a semipreparative JASCO HPLC equipped with two PU-2086 pumps, a high pressure mixer (Model MX-2080-32), a PDA detector (model MD-2018 Plus), and a Rheodyne 500 *μ*L manual injector loop was used. The software used to operate the equipment and data processing was ChromNAV version 1 : 18 : 03.

The mass spectrometry experiments were performed on LCQ Fleet equipment (Thermo Scientific) equipped with a dispositive of direct insertion of the sample via flow injection analysis (FIA). The studied matrix was analyzed by electrospray ionization (ESI), and fragmentation in multiple stages (MS^2^, MS^3^, and MS^n^) was performed at an ion trap (IT) interface. The negative mode was selected for the generation and analysis of the mass spectra for the first order (MS) and for the remaining multistage experiments under the following conditions: capillary voltage, −25 V; voltage spray, −5 kV; and capillary temperature, 275°C. A carrier gas (N_2_) with a flow of 8 arbitrary units (A.U.) was used, and the collision gas was helium (He). The track acquisition was 100–2000 *m*/*z*.* Xcalibur* version 1.3 software (Thermo Finnigan) was used to acquire and process the data.

The ^1^H- and ^13^C-NMR experiments were performed using a 300 MHz (11.7 T) nuclear magnetic resonance spectrometer (Varian Inova). CD_3_OD was used as the solvent (Sigma Aldrich).

### 2.3. Plant Material

The leaves of* T. catappa* were collected from the beach-front city of Santos,SP, with the permission of the municipality, in April 2010 and were identified by botanist Paulo Salles Penteado of University Santa Cecilia and registered (M Tomaz 01) in the Herbarium of the University of Santa Cecilia (HUSC). Authorization was obtained from IBAMA (Instituto Brasileiro do Meio Ambiente e dos Recursos Naturais Renováveis) to the number 33152.

### 2.4. Extraction

The infusion and hydroalcoholic extract were prepared according to the experimental protocol established in Biota-Fapesp Project, which this work is linked (process number 09/52237-9).

#### 2.4.1. Preparation of the Infusion of the Leaves from* T. catappa*

The leaves (1 g) of* T. catappa*, after drying, were prepared by infusion as their use in folk medicine [31].

#### 2.4.2. Preparation of the Hydroalcoholic Extract of the Leaves from* T. catappa*

The dried and powdered leaves (524.6 g) of* T. catappa* were extracted by percolation at room temperature with a mixture of EtOH : H_2_O (7 : 3, v/v). The solvents were evaporated to dryness at low pressure to give 33.1 g of the crude hydroalcoholic extract (8.7%). The extract was analyzed by FIA-ESI-IT-MS^n^ and HPLC-PDA.

#### 2.4.3. Isolation of the Fraction Containing the Punicalagin Anomers by Semipreparative HPLC

A total of 14 g of hydroalcoholic extract (7 : 3, v/v) was dissolved in H_2_O : MeOH (8 : 2, v/v) and partitioned successively with hexane (500 mL) and ethyl acetate (500 mL), giving 0.68 g (4.8%) (hexane fraction), 1.93 g (13.78%) (ethyl acetate fraction), and the 10.15 g (72.5%) (hydromethanol fraction, 8 : 2 v/v).

### 2.5. Identification of the Punicalagin Anomers and Ellagic Acid in the Hydroalcoholic Extract

Identification of the punicalagin anomers was performed using FIA-ESI-IT-MS^n^ and compared with the literature data [32], ^1^H and ^13^C NMR [33] and HPLC-PDA.

For the FIA-ESI-IT-MS^n^ assay, 10 mg of the crude hydroalcoholic extract was dissolved in 1 mL of MeOH : H_2_O (1 : 1, v/v) after using an ultrasonic bath for 5 minutes. The samples were then filtered through a 0.22 *μ*m PTFE filter, and aliquots of 20 *μ*L were directly injected into the FIA-ESI-IT-MS^n^ system.

For the HPLC-PDA a* clean-up* step was performed to remove any contaminants; the solution was purified by solid phase extraction (SPE) using Phenomenex Strata C_18_ cartridges (500 mg of stationary phase) that were previously activated with 5 mL of MeOH and equilibrated with 5 mL of MeOH : H_2_O (1 : 1, v/v). The compounds were eluted from cartridges using 1 mL of MeOH : H_2_O (1 : 1, v/v) with a final volume of 5 mL. The samples were then filtered through a 0.22 *μ*m PTFE filter and dried. The dry extract was diluted to 10 mg/mL in HPLC solvent. Aliquots of 20 *μ*L were injected directly into the HPLC-PDA with detection at 270 nm. The identification of the different compounds in the chromatographic profile of the hydroalcoholic extract was done by comparing their retention times (*t*_*r*_) and UV spectra with those obtained for the pure standards (ellagic acid, Sigma) and the compounds isolated from the hydroalcoholic extract of* T. catappa *(punicalagin anomers).

For the confirmation of the presence of ellagic acid experiments were done by coinjection. The hydroalcoholic extract (15 mg) was dissolved in 1 mL of MeOH : H_2_O (1 : 1, v/v) and subjected to a clean-up procedure using SPE cartridge RP18 (50 mg). The sample was then filtered through a PTFE filter (0.22 *μ*m) and dried at room temperature. The resulting solid was redissolved in MeOH : H_2_O to a concentration of 10 mg/mL. Two milligrams of ellagic acid standard (Sigma) was then added to this solution, and the solution was centrifuged for 2 min and filtered through a 0.22 *μ*m filter. A hydro column with a flow rate of 1 mL/min was used with a gradient of 5–60% MeOH for 60 minutes, and the absorbance at 270 nm was detected. The chromatograms of the hydroalcoholic extract were compared before and after the addition of the ellagic acid standard. From the coinjection of ellagic acid and the hydroalcoholic extract, we confirmed the presence of ellagic acid from the significant increase in the peak eluting at *t*_*r*_ 47.30 min (peak 3).

### 2.6. Conditions for Quantification of Punicalagin (Anomers) and Ellagic Acid

The quantification of analytes was performed using an external calibration standard [34, 35]. The curves were constructed using standards of the commercial ellagic acid (Sigma) and the isolated punicalagin anomers. We evaluated the content of each punicalagin anomer and the total content of punicalagins in leaves of* T. catappa*. A stock solution of 1000 *μ*g/mL ellagic acid was prepared, and dilutions to 500, 250, 125, 62.5, 15.6, 7.8, and 3.9 *μ*g/mL were made. For punicalagin, a stock solution at concentrations of 4000 *μ*g/mL of the *α*-anomer (peak 1) and 4000 *μ*g/mL of the *β*-anomer (peak 2) was prepared. From the stock solution, serial dilutions were made to reduce the concentration in 2 : 1 increments (2000, 1000, 500, 250, 125, 62.50, and 31.25 *μ*g/mL) for both the *α*- and *β*-anomers. Each concentration level was analyzed in triplicate. The mean areas of the chromatographic peaks obtained were interpolated as a function of concentration using linear regression and were used to generate the calibration curves. The linear coefficients (a) and angle (b) were obtained from the calibration curves. The correlation coefficient (*r* ^2^) and limits of detection (LOD) and quantitation (LOQ) for peaks 1 and 2 corresponding to the *α*- and *β*-anomers of punicalagin [36] were obtained. The accuracy of the HPLC method was estimated from the recovery tests. The recovery tests were performed by adding known concentrations (low, medium, and high) of the reference ellagic acid (15, 60, and 250 *μ*g/mL) and isolated punicalagin anomer (250, 500, and 2000 *μ*g/mL) standards. The average recovery value was calculated using the following formula: recovery % = [(*C*_f_/*C*_nf_ + *C*_pd_)] × 100, where *C*_f_ corresponds to fortified concentration, *C*_nf_ corresponds to the unfortified concentration and *C*_pd_ corresponds to the concentration of the added standard.

Repeatability of intra- and interday was done to determine the accuracy of the developed method. The relative standard deviation (RSD) was taken as a measure of accuracy. Repeatability of intra- and interday was determined in six replicates within the range found in the extract in a day and on three consecutive days, respectively.

### 2.7. *Salmonella*/Microsome Assay

Mutagenic activity was evaluated using the* Salmonella*/microsome assay with the* Salmonella typhimurium* tester strains TA98, TA100, TA97a, and TA102, which were kindly provided by Dr. B. N. Ames (Berkeley, CA, USA), with (+S9) and without (−S9) metabolization using the preincubation method [37]. The strains were grown overnight from frozen cultures for 12–14 h in Oxoid Nutrient Broth number 2. The metabolic activation mixture (S9 fraction), prepared from the livers of Sprague-Dawley rats treated with the polychlorinated biphenyl mixture Aroclor 1254 (500 mg/kg), was purchased from Molecular Toxicology Inc. (Boone, NC, USA) and freshly prepared before each test. The metabolic activation system consisted of 4% S9 fraction, 1% 0.4 M MgCl_2_, 1% 1.65 M KCl, 0.5% 1 M D-glucose-6-phosphate disodium, 4% 0.1 M NADP, 50% 0.2 M phosphate buffer, and 39.5% sterile distilled water. For the determination of the mutagenic activity, five different concentrations of extract (1.56 to 22.24 mg/plate), diluted in DMSO, were assayed. The concentrations of the sample were selected on the basis of a preliminary toxicity test. In all subsequent assays, the upper limit of the dose range tested was either the highest nontoxic dose or the lowest toxic dose determined in the preliminary assay. Toxicity was detected either as a reduction in the number of histidine revertants (His+) or as a thinning of the auxotrophic background (i.e., background lawn).

The various concentrations of extract to be tested were added to either 0.5 mL of 0.2 M phosphate buffer or 0.5 mL of 4% S9 mixture with 0.1 mL of bacterial culture and then incubated at 37°C for 20–30 min. Next, 2 mL of top agar was added, and the mixture was poured onto a plate containing minimal agar.

The plates were incubated at 37°C for 48 h, and the His+ revertant colonies were counted manually. All experiments were analyzed in triplicate. The results were analyzed with the statistical software package Salanal 1.0 (U.S. Environmental Protection Agency, Monitoring Systems Laboratory, Las Vegas, NV, from the Research Triangle Institute, RTP, NC, USA), adopting the Bernstein et al. [38] model. The data (revertants/plate) were assessed by analysis of variance (ANOVA) followed by linear regression. The mutagenic index (MI), which is the average number of revertants per plate with the test compound divided by the average number of revertants per plate with the negative (solvent) control, was also calculated for each concentration tested. A sample was considered mutagenic when a dose-response relationship was detected and a twofold increase in the number of mutants (MI ≥ 2) was observed for at least one concentration [39]. The standard mutagens used as positive controls in experiments without the S9 mix were 4-nitro-*o*-phenylenediamine (10.0 *μ*g/plate, TA98), sodium azide (1.25 *μ*g/plate, TA100), mitomycin (0.5 *μ*g/plate, TA102) and 2-anthramine (1.25 *μ*g/plate, TA98, TA100), and 2-aminofluorene (10.0 *μ*g/plate, TA102) in the presence of S9. DMSO served as the negative (solvent) control.

## 3. Results and Discussion

**Figure 1:**
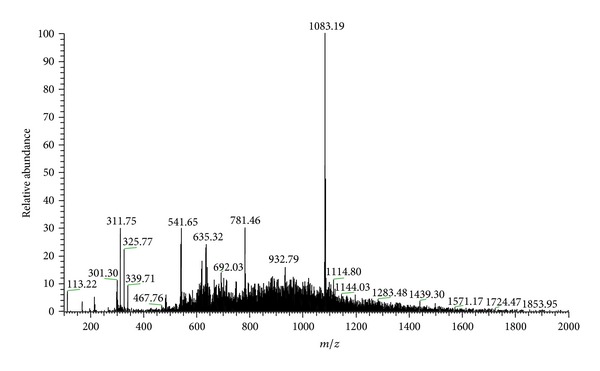
First-order mass spectrum of the hydroalcoholic extract from the leaves of* T. catappa* in the negative mode. Range of ions with *m*/*z* 100–2000 Da.

**Figure 2:**
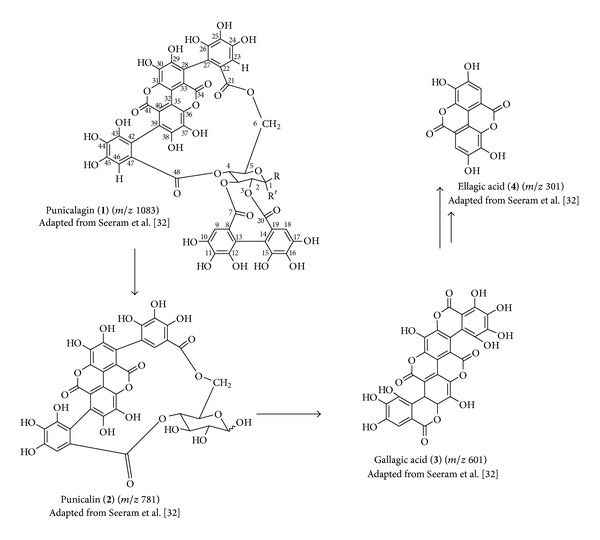
Punicalagin (**1**), punicalin (**2**), gallagic acid (**3**), and ellagic acid (**4**). Adapted from Seeram et al. [32].

The analysis of mass spectra of the hydroalcoholic extract (Figure 1) showed the precursor ions [M-H]^−^ *m*/*z* 1083 (punicalagin), *m*/*z* 781 (punicalin), *m*/*z* 601 (gallagic acid), and *m*/*z* 301 (ellagic acid) [32]. Second-order fragmentation (MS^2^) confirmed the presence of the metabolites shown in the scheme of Figure 2 for substances** 1**–**4** (Table 1). Seeram et al. [32] studying the commercial juice industry of the pomegranate husk identified the same fragmentation of these same compounds.

**Figure 3:**
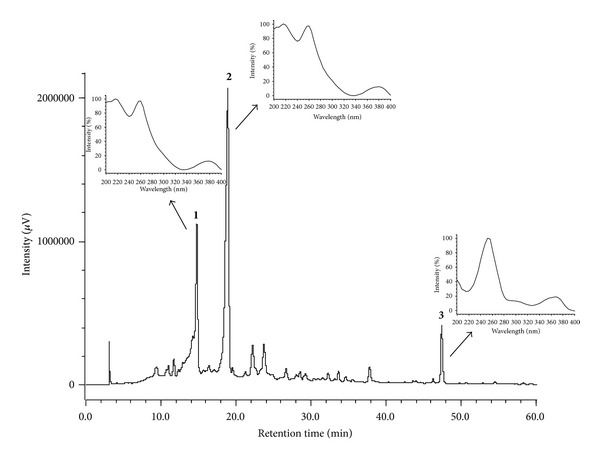
Chromatogram of hydroalcoholic extract of the leaves of* T. catappa* dissolved in MeOH : H_2_O (1 : 1 v/v), at 10 mg/mL. The method utilized a hydro column at a flow rate of 1 mL/min with a gradient of 5–60% MeOH for 60 min HPLC-PDA (Jasco), 270 nm. The UV spectra from *t*_*r*_ 14.75 (peak 1) min and at *t*_*r*_ 18.86 min (peak 2), characteristic of the *α*- and *β*-anomers of punicalagin (**1**,** 2**) and ellagic acid (**3**).

**Figure 4:**
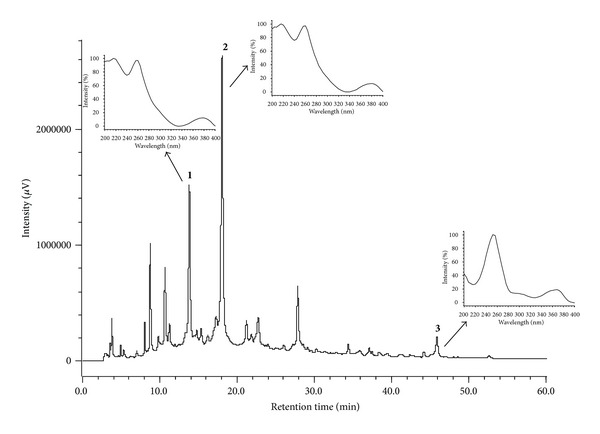
Chromatogram of infusion of the leaves of* T. catappa*. Gradient 5–60% MeOH, 60 min, 270 nm. Peaks** 1** and** 2** = punicalagins,** 3** = ellagic Acid. The UV spectra of peak 1, eluting at *t*_*r*_ 14.70 min and peak 2 at *t*_*r*_ 18.70 min, characteristic of the *α*- and *β*-punicalagin anomers. Peak 3, at *t*_*r*_ 47.30 min.

The chromatographic profile by HPLC-PDA of the hydroalcoholic extract and the infusion are shown in Figures 3 and 4. In the chromatogram, three major peaks (**1**–**3**) eluting at *t*_*r*_ 14.75 min, *t*_*r*_ 18.80 min, and *t*_*r*_ 47.30 min are observed. Figures 3 and 4 show the UV region of the spectrum obtained using a PDA detector for the peaks eluting at *t*_*r*_ 14.75 min (peak 1) and *t*_*r*_ 18.80 min (peak 2). The data confirm the presence of both punicalagin anomers with absorption maxima at *λ*_max⁡_ 218, 260, and 379 nm. The compound eluting at *t*_*r*_ 47.30 min (peak 3) corresponds to ellagic acid with *λ*_max⁡_ values of 250, 306, and 368 nm, as confirmed by coinjection experiments with a standard analyte (Figures 3 and 4). The data obtained for UV anomers punicalagin and ellagic acid are consistent with those found in the literature [40] and are characteristic of gallagyl chromophore [41].

**Figure 5:**
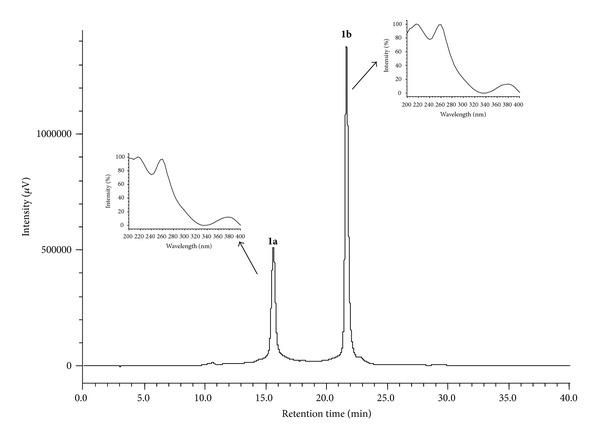
Chromatogram peak** 1a** (*α*-punicalagin) and peak** 1b** (*β*-punicalagin) collected from H_2_O : MeOH (8 : 2 v/v) at 10 mg/mL of the leaves of* T. catappa*, eluting at *t*_*r*_ 15.29 min (**1a**) and *t*_*r*_ 21.07 min (**1b**). The flow rate was 1 mL/min with a gradient 5–30% MeOH for 30 minutes using a hydro column. HPLC-PDA (Jasco), 270 nm. The UV spectra of peak 1, eluting at *t*_*r*_ 15.29 min (**1a**) and at *t*_*r*_ 21.07 (**1b**) min, characteristic of the *α*- and *β*-punicalagin anomers.

Peaks** 1** and** 2** (Figure 3) were partitioned (item 2.4.3), collected separately, and analyzed by HPLC-PDA (Figure 5). The analysis of the peaks by HPLC-PDA with *t*_*r*_ 14.75 min and 18.80 min, respectively, display an interesting behavior, as they resolve into two peaks with distinct retention times when analyzed under the same conditions to obtain peaks at similar retention times with the same UV spectra. This indicates that the compounds are the mixture of anomers (*α*- and *β*-punicalagin). These compounds have absorption maxim at *λ*_max⁡_ 218, 260, and 379 nm and are characteristic of the chromophore gallagyl [41]. For peak** 1**, the HPLC-PDA analysis shows the presence of two peaks (**1a** and** 1b**) with retention times of 15.29 min and 21.07 min, respectively (Figure 5).

In solution, punicalagin rapidly interconverts between the *α*- and *β*-anomers (Figure 2, compound** 1**). The ^13^C NMR (Table 2) spectrum reveals the presence of two anomeric carbons at *δ* 89.87 and *δ* 93.77, corresponding to punicalagins *α* (present in lesser proportion in peak 1) and *β* (present in a greater proportion in peak 2), respectively. The stereochemistry at the anomeric position can be easily determined by measuring the coupling constant between the anomeric proton H-1 and the adjacent proton H-2 in the ^1^H NMR spectrum. A large coupling constant (*J*_H-1,H-2_ > 5 Hz) generally indicates a *β*-glycosidic configuration (substituent in the equatorial orientation), whereas a small coupling constant (*J*_H-1,H-2_ = 0–5, Hz) indicates an *α*-glycosidic configuration (substituent in the axial orientation). The chemical shift and the coupling constant corresponding to the *α*-anomer is *δ* 5.08 (*J* = 3.5 Hz) and for the *β*-anomer is *δ* 4.66 (*J* = 8.0 Hz). NMR data of the isolated anomers (*α*- and *β*-punicalagin) are shown in the Table 2 (enumeration of carbons and hydrogens in Figure 2) compared with data found in the literature [33]. The deprotonated molecule generated in the FIA-ESI-IT-MS [M-H]^−^ at *m*/*z* 1083 (1084 MW) (Table 1) and the UV spectra exhibited by *α*- and *β*-punicalagin confirmed this identification (Figure 5).

The linear regression analysis of the peak area for *α*- and *β*-punicalagin, the total punicalagins, and ellagic acid are showed in the Table 3. Thus, it becomes necessary to use sufficient number of standard solutions to adequately define the relationship between concentration and response [42]. These results demonstrate the linearity of the method over the analyzed concentration range.

The recovery percentages for ellagic acid and total punicalagins were found to be 87% and 86%, respectively (Table 4). The results were presented as mean and standard deviation of the percentages recovered. The results showed that the accuracy of the method is very good for all analytes (RSD < 2%). The relative standard deviations also showed that the process of sample preparation is reproducible [43].

The relative standard deviations (RSDs) for repeatability testing intra- and interday analyses are shown in Table 5. The result is presented as relative standard deviation from the average. The method showed excellent repeatability, with all RSD values lower than 1% [44].

Table 6 shows the mean number of revertants/plate (M), the standard deviation (SD), and the mutagenic index (MI) after the treatments with the* T. catappa *hydroalcoholic extract, observed in* S. typhimurium *strains TA98, TA100, TA102, and TA97a, in the presence (+S9) and absence (−S9) of metabolic activation.

In the TA98 strain, the* T. catappa* hydroalcoholic extract did not induce any increase in the number of revertant colonies under the conditions used in this study. The other strains were more sensitive to the toxic effects of extract, and it was thus necessary to decrease the doses.

Only in the absence of the external metabolizing system, S9 mix, in the* S. typhimurium* strains TA100 and TA97a, the* T. catappa* hydroalcoholic extract induced an increase in the number of revertant colonies relative to the negative control, with a MI higher than 2.0 at the concentration of 3.12 mg/plate in strain TA100 and 9.38 mg/plate in strain TA97a, indicating the direct mutagenic activity for these strains.

In the TA102 strain (+S9 and –S9), the* T. catappa* hydroalcoholic extract shows signs of mutagenicity, with MI values approximately 2.

The* S. typhimurium* test strain TA97a detects frameshift mutations in C-C-C-C-C-C; +1 cytosine and TA98 frameshift in DNA target-C-G-C-G-C-G-C-G, the* S. typhimurium *tester strain TA100 is capable of revealing base-pair substitution point mutations and the* S. typhimurium* tester strain TA102 is normally used to detect cross-linking agents and base-pair substitution mutations [39]. Thus, according to the strains involved, the* T. catappa *hydroalcoholic extract mainly induces substitution of base pairs (TA100) and frameshift mutations (TA97a).

Ko et al. [17] evaluated the toxicity and mutagenicity of supercritical carbon dioxide (SC-CO_2_) extracts of* T. catappa* leaves at a dose of 0.5 mg/plate in the strain TA100 (detect base-pair substitution) and observed absence of mutagenic effect. The absence of mutagenic effect was also observed for TA102 and TA98 when the ethanolic extract was evaluated.

These studies show that mutagenic activity of* T. catappa* in the Ames test can only be observed when measured at high concentrations. The results of this study are consistent with a review of the literature indicating that high concentrations of tannins (15 and 60 mM) can cause DNA damage. These data corroborate the observations of Gupta et al. [45], which indicated that all tannins result in polyphenol toxicity at high concentrations [46].

## 4. Conclusions

In the hydroalcoholic extract of* T. catappa*, tannins were the most abundant compounds observed, and the anomers *α*- and *β*-punicalagin were the major compounds. Moreover, considering that the extract is a complex mixture of several unknown organic compounds, the mutagenicity observed may be explained in part by a synergy between compounds present in the extract.

The dosage and type of tannin involved are critical for these effects. Thus, the results obtained in this study are useful for better understanding the pharmacological activities of* T. catappa*. However, considering the mutagenic effects observed in this study, this plant should be used cautiously for medicinal purposes.

Based on the obtained results, it can be concluded that the developed method (HPLC-PDA) is suitable for its purpose, namely, the determination of anomers punicalagin and ellagic acid in extract of* T. catappa*.

## Figures and Tables

**Table 1:** Identification of substances in *T. catappa* by FIA-ESI-IT-MS^n^.

[M–H]^−^	MS^n^ ions	Identification
1083	781 [M-152-152-H]^−^ 601 [M-152-152-180-H]^−^	Punicalagin **(1)**
781	601 [M-180-H]^−^	Punicalin **(2)**
601	409 [M-191-H]^−^	Gallagic acid **(3)**
301	257 [M-44-H]^−^ 229 [M-44-28-H]^−^	Ellagic acid **(4)**

**Table 2:** Chemical shifts (^1^H and ^13^C) of the *α*- and *β*-punicalagins in CD_3_OD isolated from *T. catappa*.

Position	*α*-punicalagin			*β*-punicalagin		
	*δ* (^1^H)	Multiplicity (Hz)	*δ* (^13^C)	*δ* (^1^H)	Multiplicity (Hz)	*δ* (^13^C)
1	5.08	d(3.5)	89.87	4.66	d(8.0)	93.77
2	4.87	dd(3.0, 8.0)	73.53	4.62	dd(3.0, 8.1)	75.48
3	5.20	t(9.3)	76.08	4.92	t(10.0)	78.78
4	4.78	t(9.0)	70.23	4.85	t(10.0)	74.88
5	3.23	t(9.0)	66.33	2.57	dd(9.9, 11.0)	71.58
6a	2.10	d(9.9)	63.63	4.05	d(9.9)	63.03
6b	4.09	dd(7.0, 7.0)	63.63	4.10	d(7.8)	63.03
9	6.52	s	105.46	6.54	s	106.21
18	6.66	s	107.41	6.68	s	106.21
23	6.73	s	108.76	6.77	s	109.51
46	6.94	s	110.11	6.97	s	110.71

**Table 3:** Parameters for quantification of the isolated compounds.

	Linear coefficient (*a*)	Slope (*b*)	Correlation coefficient	LOD *µ*g/mL	LOQ *µ*g/mL
Ellagic acid	21753	71514	0.998	1.00	3.04
*α*-punicalagin	37326	11861	0.997	10.38	31.46
*β*-punicalagin	44760	16830	0.999	8.77	26.59
Total punicalagins	**82086**	**28691**	**0.998**	**9.44**	**28.61**

**Table 4:** Determining the accuracy of the method for the determination of each of the substances quantified.

Substances investigated	Repetition (%)			Recovery mean	DP	RSD (%)
	**1**	**2**	**3**			
Ellagic acid	90.4	87.75	84.52	87.56	2.94	0.03
Total punicalagins	86.9	86.26	85.86	86.34	0.52	0.01

RSD (%) = RSD: relative standard deviation (100 × SD/mean).

**Table 5:** Repeatability test results for each substance quantified.

Substances investigated	Intraday (*n* = 6)			Interday (*n* = 6)		
	Mean (%)	DP	RSD (%)	Mean (%)	DP	RSD (%)
Ellagic acid	87.44	0.12	0.001	87.53	0.19	0.002
Total Punicalagins	86.34	0.19	0.002	86.19	0.17	0.002

RSD (%) = RSD: relative standard deviation (100 × SD/mean).

**Table 6:** Mutagenic activity expressed as the mean and standard deviation of the number of revertants/plate and mutagenic index (in parenthesis) in *Salmonella typhimurium* TA98, TA100, TA97a, and TA102 strains treated with hydroalcoholic extract of *T. catappa* at various doses, with (+S9) or without (−S9) metabolic activation.

	Treatments	Number of revertants (M ± SD)/ plate and MI								
		TA98			TA100		TA102		TA97a	
	mg/plate	−S9	+S9	mg/plate	−S9	+S9	−S9	+S9	−S9	+S9
Terminalia catappa	0.0^a^	16 ± 3	68 ± 1	0.0^a^	93 ± 6	87 ± 4	352 ± 30	435 ± 43	172 ± 2	99 ± 1
	2.78	17 ± 6 (1.1)	75 ± 7 (1.1)	1.56	91 ± 2 (1.0)	88 ± 9 (1.0)	461 ± 11* (1.3)	481 ± 23 (1.1)	223 ± 3* (1.3)	127 ± 3 (1.3)
	5.56	21 ± 3 (1.3)	75 ± 7 (1.1)	3.12	188 ± 19** (2.0)	90 ± 6 (1.0)	514 ± 16* (1.5)	668 ± 75* (1.5)	252 ± 12* (1.5)	102 ± 7 (1.0)
	11.12	18 ± 5 (1.1)	57 ± 1 (0.8)	6.25	210 ± 24** (2.3)	91 ± 6 (1.0)	653 ± 22* (1.9)	689 ± 57* (1.6)	287 ± 14* (1.7)	95 ± 12 (1.0)
	16.68	14 ± 3 (0.9)	75 ± 4 (1.1)	9.38	40 ± 4 (0.4)	95 ± 1 (1.1)	563 ± 32* (1.6)	728 ± 28* (1.7)	337 ± 19** (2.0)	93 ± 10 (0.9)
	22.24	2 ± 1 (0.1)	68 ± 10 (1.0)	12.5	Nd	88 ± 4 (1.0)	648 ± 32* (1.8)	621 ± 23* (1.4)	397 ± 19** (2.3)	83 ± 2 (0.8)
	Ctrol+	720 ± 63	500 ± 57	Ctrol+	1225 ± 75	700 ± 80	1143 ± 53	1309 ± 38	1426 ± 67	700 ± 45

*P < 0.05 (ANOVA); **P < 0.01 (ANOVA), 0^a^: Negative control: dimethyl sulfoxide (DMSO—75 *μ*L/plate); Positive Control: 4-nitro-*o*-phenylenediamine (10.0 *μ*g/plate—TA98 and TA97a); sodium azide (1.25 *μ*g/plate—TA100); mitomycin (0.5 *μ*g/plate—TA102), in the absence of S9, and 2-anthramine (1.25 *μ*g/ plate—TA98, TA100, TA97a); 2-aminofluorene (10.0 *μ*g/plate—TA102), in the presence of S9. Nd: not determined.
